# Subclinical inflammation in pediatric familial Mediterranean fever: predictors and follow-up results of a retrospective single-center cohort study

**DOI:** 10.1007/s10067-026-08133-8

**Published:** 2026-04-24

**Authors:** Onur Bahçeci, Fatma Aydin, Özen Taş Aslan, Doğacan Sarisoy, Elif Erorhan, Zeynep Birsin Özçakar

**Affiliations:** 1 Department of Pediatrics, Division of Pediatric Rheumatology, Ankara University School of Medicine, Ankara, Turkey; 2 Department of Pediatrics, Division of Pediatric Nephrology, Ankara University School of Medicine, Ankara, Turkey

**Keywords:** Colchicine, Familial Mediterranean fever (FMF), IL-1 inhibitors, Subclinical inflammation

## Abstract

**Introduction/objectives:**

Familial Mediterranean fever (FMF) is the most common monogenic autoinflammatory disease in childhood. Subclinical inflammation is defined as persistently elevated acute phase reactants despite the absence of clinical attacks. The aim of this study was to determine the frequency of subclinical inflammation and its clinical predictors in pediatric FMF patients, and to assess treatment responses following therapeutic adjustments.

**Methods:**

Medical records of FMF patients followed between January 2011 and February 2024 were retrospectively reviewed. Patients were grouped based on the presence of subclinical inflammation, and clinical features, genetics, and treatment responses were compared to identify risk factors for subclinical inflammation.

**Results:**

A total of 572 pediatric FMF patients were included, of whom 89 (15.6%) exhibited subclinical inflammation. Patients with subclinical inflammation had significantly earlier disease onset, longer disease duration, numerically more frequent comorbid conditions, and more biallelic exon 10 mutations (*p* < 0.05). In addition, arthritis and higher disease severity scores were detected as independent predictors of subclinical inflammation. Regarding treatment, 45% of patients with subclinical inflammation achieved resolution after colchicine dose adjustments. However, 55% required biologic therapies. Among biologic-treated patients, 87.7% showed normalization of inflammatory markers, whereas 12.3% continued to exhibit persistent inflammation; all of these patients had comorbid conditions.

**Conclusion:**

Subclinical inflammation is common in pediatric FMF patients. Our findings suggest that earlier disease onset, biallelic exon 10 mutations, arthritis, and higher disease severity scores are associated with persistent inflammation. Colchicine dose optimization effectively controls inflammation in nearly half of these patients, while biologic therapies, particularly IL-1 inhibitors, are effective for the remaining cases.

**Key points:**

• *Subclinical inflammation is common in pediatric FMF patients.*

• *Early disease onset, biallelic exon 10 mutations, arthritis, and higher disease severity scores are associated with subclinical inflammation.*

• *Colchicine dose adjustments are effective for nearly half of patients, while biologic therapies, particularly IL-1 inhibitors, are needed for the rest.*

## Introduction

Familial Mediterranean fever (FMF) is one of the earliest known and most prevalent autoinflammatory condition. It is a genetically inherited periodic fever syndrome, accompanied by recurrent, self-limiting febrile episodes and polyserositis. This disorder is predominantly observed among ethnic groups originating from the Mediterranean region, including individuals of Turkish, Armenian, Jewish, and Arab descent [1].

The Mediterranean fever (*MEFV*) gene, located on the short arm of chromosome 16, was first identified in 1997 by the International and French Consortium as the genetic determinant of FMF. It encodes “pyrin” a 781-amino acid protein that plays a critical role in the regulation of inflammation and apoptosis [2, 3]. Elevated interleukin (IL)−1β levels contribute to the inflammatory features of FMF, and targeting this pathway has opened new therapeutic possibilities [4]. Colchicine has been the cornerstone of FMF treatment for over four decades, effectively managing clinical symptoms, reducing inflammatory markers, and preventing the development of amyloidosis. Nevertheless, approximately 5–10% of patients exhibit resistance to colchicine. In such cases, IL-1β–targeted therapies have shown promising outcomes [5].

In FMF, attacks are typically short-lived, lasting between 1 and 4 days, and are associated with significant elevations in acute phase reactants [6]. While normalization of inflammatory markers is anticipated during asymptomatic intervals, a subset of patients continues to exhibit persistently elevated levels of inflammatory parameters such as C-reactive protein (CRP), erythrocyte sedimentation rate (ESR), white blood cell count (WBC), and serum amyloid A (SAA), indicating the presence of subclinical inflammation even in the absence of overt clinical symptoms [7]

Subclinical inflammation plays a crucial role in the long-term progression of FMF, contributing to complications such as normocytic-normochromic anemia and growth retardation in children, reduced bone mineral density, and the development of AA amyloidosis [8–11]. These complications are often exacerbated in colchicine-resistant patients and those with high-risk genotypes such as homozygous M694V, underscoring the significance of genotype–phenotype correlations in FMF pathogenesis [12]. Emerging data further emphasize the influence of both genetic and environmental factors on disease variability, highlighting the need for personalized treatment strategies tailored to subclinical disease activity [13]. However, despite its recognized clinical significance, there remains a limited number of studies focusing on the risk factors, treatment approaches, and persistence of subclinical inflammation following therapy in FMF patients.

The aim of this study was to investigate the frequency of subclinical inflammation in pediatric patients with FMF, to evaluate the associated factors and the course of the condition, and to identify the risk factors for subclinical inflammation.

## Materials and methods

This retrospective single-center cohort study included pediatric patients diagnosed with familial Mediterranean fever (FMF) according to the Yalçınkaya–Ozen criteria and followed at our pediatric rheumatology center between January 2011 and February 2024 [6]. Medical records were reviewed for demographic, clinical, laboratory, genetic, and treatment-related data. Patients were eligible for inclusion if they were younger than 18 years of age at diagnosis, had regular clinical and laboratory follow-up for at least 1 year, and had available follow-up data during attack-free periods.

Demographic characteristics (age, gender), clinical parameters (family history, parental consanguinity, positive family history, age at disease onset, diagnosis delay, follow-up duration, attack findings, attack frequency per year, etc.), and comorbid inflammatory diseases were all recorded. *MEFV* gene mutation analysis was performed using standardized molecular methods. At least six predominant mutations (p.M694V, p.M680I, p.M694I, p.V726A, p.K695R, and p.E148Q) in the *MEFV* gene have been identified. Patients were categorized according to their mutations as follows: single exon 10 mutation (heterozygous), biallelic exon 10 mutations (homozygous or compound heterozygous), and other non-exon 10 variants. Treatment details (colchicine dose, anti-IL-1 treatment, anti-TNF treatment) and laboratory data (CRP, ESR, fibrinogen, WBC, hemoglobin) were collected from patient records. Disease severity was measured using the International Severity Scoring System for FMF (ISSF), which has a maximum score of 10. Based on this scale, disease severity is categorized as severe (≥ 6), moderate (3–5), or mild (≤ 2) [14]. The nutritional status of patients was assessed by the calculation of body mass index (BMI) *Z*-scores, a globally accepted method by the World Health Organization (WHO) to assess growth and nutritional status in children and adolescents [15].

Colchicine resistance was characterized by the recurrence of clinical attacks (at least once per month, on average, over a 3-month period) or by the persistence of elevated inflammatory markers, despite receiving the maximum tolerated dose of colchicine for at least six months [5].

Subclinical inflammation was defined as persistently elevated CRP levels during attack-free periods, which were defined as a minimum of 10 days after the last attack. This had to be observed in more than 75% of a patient’s routine follow-up assessments [16, 17]. Laboratory evaluations performed during overt FMF attacks or visits suggestive of acute infection or other acute inflammatory conditions were not considered for the definition of subclinical inflammation. As part of routine clinical care in our outpatient clinic, all FMF patients are systematically assessed at each follow-up visit for recent attacks and potential infectious exposures (in the patient or close contacts). A comprehensive physical examination is conducted, and attack and infection status are recorded. This standardized evaluation facilitates accurate interpretation of acute phase reactants during follow-up.

Patients were divided into two groups based on the presence or absence of subclinical inflammation. We evaluated potential differences in clinical presentation, genetics and treatment response between groups, and investigated risk factors for subclinical inflammation. We also reviewed patient progress in terms of subclinical inflammation.

## Statistical analysis

Statistical analysis was performed using IBM SPSS Statistics version 25 (IBM Corp. Armonk, NY, USA). The distribution of continuous variables was assessed through visual (histogram, Q-Q plots) and analytical methods (Kolmogorov–Smirnov and Shapiro–Wilk tests). Continuous variables were summarized as medians with interquartile ranges (IQR), and categorical variables as numbers and percentages. Intergroup comparisons for continuous variables were conducted using the Mann–Whitney *U* test, while categorical variables were analyzed using the chi-square test or Fisher’s exact test, as appropriate. Univariate logistic regression was performed to identify variables associated with subclinical inflammation. Variables associated with subclinical inflammation in univariate analyses and/or considered clinically relevant were evaluated for inclusion in the multivariable logistic regression model. Odds ratios (ORs) and 95% confidence intervals (CIs) were calculated. Variables with high collinearity (correlation coefficient > 0.60) were carefully reviewed, and the clinically less relevant or statistically weaker variables were excluded. Model adequacy was evaluated using the Hosmer–Lemeshow test. Cases with missing data were excluded from the relevant analyses. No imputation was performed. A *p* value < 0.05 was considered statistically significant.

This study was approved by the local ethics committee (approval number: 2025000463–3/11.08.2025) and was conducted in accordance with the principles of the Declaration of Helsinki.

## Results

A total of 572 pediatric patients diagnosed with FMF were included in this study. Among these, 89 (15.6%) of them exhibited subclinical inflammation. The median (IQR) age at disease onset was significantly lower in the subclinical inflammation group compared to the non-inflammation group (*p* = 0.038). The median (IQR) disease duration time was longer in patients with subclinical inflammation (*p* < 0.001). Parental consanguinity was more common among patients with subclinical inflammation (*p* = 0.024). *MEFV* gene analysis revealed a significantly higher frequency of biallelic exon 10 mutations in the subclinical inflammation group (*p* < 0.001). Comorbid diseases were more common (*not significantly*) in the subclinical inflammation group (*p* = 0.055), particularly inflammatory bowel disease (IBD), chronic non-bacterial osteomyelitis (CNO), and juvenile idiopathic arthritis (JIA). In our cohort, patients with subclinical inflammation had lower hemoglobin levels (*p* < 0.001) and lower BMI *Z*-scores (*p* < 0.001). Renal amyloidosis was observed in two patients.

Comparison of clinical and laboratory features of the 2 groups with and without subclinical inflammation is given in Table 1.

**Table 1:** Comparison of clinical and laboratory parameters of patients with and without subclinical inflammation

	Patients with subclinical inflammation ***n*** = 89 (%), median (IQR)	Patients without subclinical inflammation ***n*** = 483 (%), median (IQR)	***p value***
**Gender (female)**	45 (50.6)	243 (50.3)	0.96
**Age at disease onset (years)**	5.5 (6)	6.5 (6)	**0.038**
**Diagnosis delay (years)**	1 (2)	1 (3)	0.88
**Duration of disease (years)**	7 (5)	6 (5)	** < 0.001**
**Age at the last follow-up (years)**	15 (6)	14 (8)	0.075
**Positive family history for FMF**	61 (68.5)	294 (60.9)	0.17
**Parental consanguinity**	30 (33.7)	109 (22.6)	**0.024**
**Family history of amyloidosis**	11 (12.4)	33 (6.8)	0.073
**MEFV mutations** One allele exon 10 Two allele exon 10 Others	10 (11.2) 75 (84.3) 4 (4.5)	163 (33.7) 250 (51.8) 70 (14.5)	** < 0.001**
**Comorbid disease presence** Inflammatory bowel disease Chronic non-bacterial osteomyelitis Juvenil idiopathic arthritis Vasculitis Behcet’s disease	13 (14.6) 5 (5.6) 3 (3.4) 5 (5.6) 0 (0) 0 (0)	36 (7.5) 8 (1.7) 5 (1) 5 (1) 15 (3.1) 3 (0.6)	**0.055**
**Clinical findings**			
Abdominal pain	58 (65.2)	292 (60.5)	0.40
Chest pain	8 (9)	41 (8.5)	0.87
Fever	59 (66.3)	241 (49.9)	**0.004**
Erysipelas-like erythema	3 (3.4)	3 (0.6)	0.051
Arthritis	13 (14.6)	13 (2.7)	** < 0.001**
Arthralgia	14 (15.7)	46 (9.5)	0.079
Pericarditis	1 (1.1)	0 (0)	0.15
**Frequency of attacks/year**	2 (3)	1 (2)	** < 0.001**
**Laboratory measurements**			
White blood cell count (× 10^9^/L), median (IQR)	13260 (5065)	7194 (2700)	** < 0.001**
Hemoglobin (g/dL) median (IQR)	12.6 (1.9)	13.2 (1.8)	** < 0.001**
C-reactive protein (mg/L), median (IQR)	21.1 (19.9)	1.1 (2.7)	** < 0.001**
Erythrocyte sedimentation rate (mm/h), median (IQR)	21.4 (18.9)	7 (8)	** < 0.001**
Fibrinogen (mg/dL), median (IQR)	5.09 (1.15)	2.92 (1.03)	** < 0.001**
**ISSF score** Mild (≤ 2 points) Moderate-severe (≥ 3 points)	41 (46.1) 48 (53.9)	404 (83.6) 79 (16.4)	** < 0.001**
**Dose of colchicine (mg/m**^**2**^**/day)**	1.43 (0.17)	1 (0.02)	** < 0.001**
**Anti IL-1 treatment**	35 (39.3)	2 (0.4)	** < 0.001**
**Anti TNF treatment**	14 (15.7)	3 (0.6)	** < 0.001**
**BMI *Z*-score**	−0.44 (1.04)	−0.2 (1.23)	**0.031**

*IQR*, interquartile range (Q1–Q3); *BMI*, body mass index; *FMF*, Familial Mediterranean fever; *MEFV*, Mediterranean fever gene; *ISSF*, International Severity Score for FMF

^*^Statistically significant results are in bold

In multivariate logistic regression analysis, longer disease duration, presence of biallelic exon 10 mutations, and presence of arthritis were independent risk factors for subclinical inflammation. In addition, higher disease severity score was also an independent predictor of subclinical inflammation (Table 2).

**Table 2:** Risk factors associated with subclinical inflammation in FMF patients: univariate and multivariate logistic regression analyses

**Factors**	**Univariate analysis**		**Multivariate analysis**	
	**OR (95% CI)**	***p* value**	**OR (95% CI)**	***p* value**
Age at disease onset	0.94 (0.89–1.00)	0.052	0.94 (0.87–1.02)	0.15
Duration of disease	1.12 (1.05–1.19)	** < 0.001**	1.09 (1.01–1.17)	**0.018**
Attack frequency/year	1.21 (1.11–1.32)	** < 0.001**	1.10 (0.99–1.22)	0.068
Presence of biallelic exon 10 mutations	3.42 (1.21–9.63)	**0.02**	4.20 (1.33–13.25)	**0.014**
Arthritis	6.17 (2.75–13.81)	** < 0.001**	8.01 (3.17–20.68)	** < 0.001**
BMI *Z*-score	0.74 (0.57–0.94)	**0.017**	0.77 (0.6–1.02)	0.061
ISSF score	5.98 (3.69–9.69)	** < 0.001**	5.28 (3.00–9.31)	** < 0.001**

*BMI*, body mass index; *ISSF*, International Severity Score for FMF; *CI*, confidence interval; *OR*, odds ratio

**Fig. 1:**
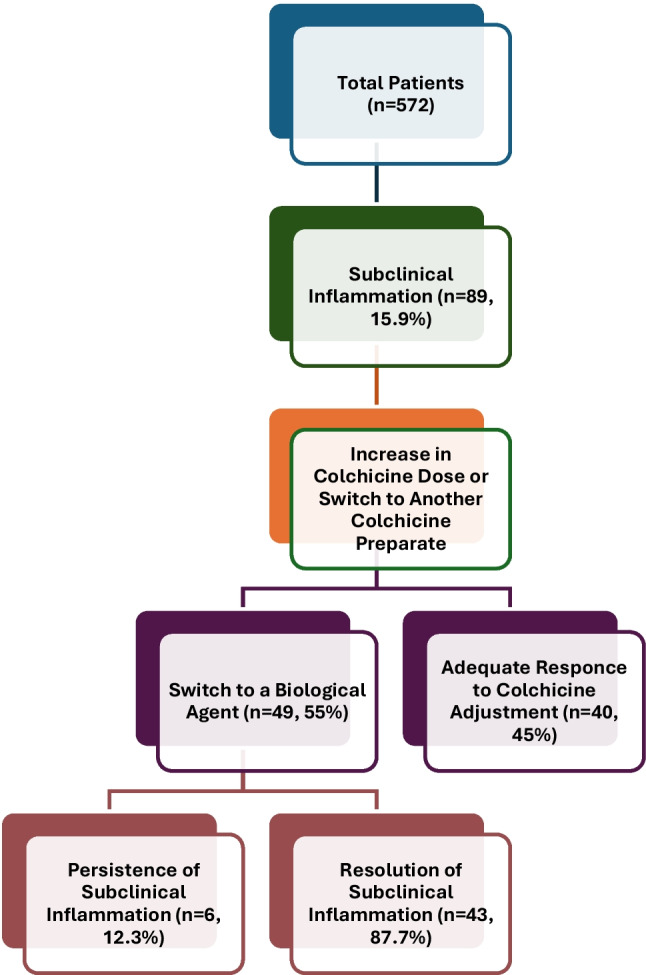
Treatment courses of FMF patients with subclinical inflammation

Among 89 patients with subclinical inflammation, 40 (45%) showed resolution of inflammation following adjustment of the colchicine dose or switching between different colchicine preparations. Colchicine was well tolerated in our cohort, and no colchicine-related adverse effects requiring treatment discontinuation were observed. However, 49 patients (55%) were found to have persistent subclinical inflammation and required biologic therapy. Of these patients, 35 received anti-IL-1 treatment, while 14 were treated with anti-TNF agents. Twelve patients who received anti-TNF therapy had coexisting comorbidities associated with FMF, which influenced the selection of the biologic agent (FMF-associated sacroiliitis in 3 patients, Crohn’s disease in 1 patient, uveitis in 1 patient, JIA in 5 patients, and CNO in 1 patient, SAPHO and IgA nephropathy in 1 patient). Additionally, in two patients with amyloidosis, anti-TNF therapy was administered during periods when anti-IL-1 treatment was not accessible in our country. Following biologic therapy, subclinical inflammation resolved in 43 patients (87.7%), while six patients (12.3%) continued to exhibit persistent laboratory evidence of inflammation despite treatment. All six of these patients had comorbidities. Treatment courses of FMF patients are summarized in the flowchart (Fig. 1).

## Discussion

In this study, we found that approximately one-sixth of our FMF patients exhibited subclinical inflammation during asymptomatic intervals, suggesting that clinically silent disease activity may persist in a considerable proportion of pediatric cases. Earlier age at disease onset, carrying biallelic exon 10 mutations, presence of arthritis, and higher ISSF scores seem to be related to subclinical inflammation. The resolution of the inflammation was achieved through either an increase in the colchicine dose or a change in the preparation in approximately 50% of cases. The remaining patients required biological therapies, predominantly IL-1 inhibitors, which resulted in substantial normalization of inflammatory markers. Only 6 patients had persistent subclinical inflammation due to the presence of concomitant inflammatory diseases.

Familial Mediterranean fever is an autoinflammatory disease characterized by recurrent and spontaneously terminating attacks. Despite the variability of attacks among patients, the presence of subclinical inflammation, characterized by its silent yet biochemically persistent nature, can be detected in some patients during attack-free periods that are deemed clinically asymptomatic [18]. The main aim of treatment protocols for FMF is to prevent attacks and control subclinical inflammation, thereby reducing the risk of long-term complications such as amyloidosis [19]. In adult FMF patients, subclinical inflammation has been reported in approximately 15% of cases [17]. In two different pediatric studies, it was found that 12% and 20% of pediatric FMF patients exhibited persistent inflammation during attack-free periods [13, 16]. In the present study, this rate was found to be similar, with subclinical inflammation detected in 15.6% of our FMF patients. These findings suggest a consistent prevalence of subclinical inflammation across different cohorts, indicating that such inflammation is a common feature in both pediatric and adult FMF populations, warranting further attention in clinical management.

Subclinical inflammation is particularly prevalent in patients with early-onset FMF. Early-onset disease is generally associated with more severe disease activity and an increased inflammatory burden [17, 20]. Gezgin Yıldırım et al. demonstrated that the age of onset was significantly younger in pediatric FMF patients with persistent inflammation [16]. Similarly, in our study, the median age at disease onset was significantly lower in the group with subclinical inflammation than in the group without (5.5 vs. 6.5 years).

Arthritis is the most common feature of FMF attacks after peritonitis, affecting approximately 50% of patients, particularly in children. Arthritis has been associated with a severe disease course in FMF patients [17, 21, 22]. Notably, a significantly higher prevalence of arthritis was found in FMF patients with subclinical inflammation than in those without (66% vs 38.8%) in an adult study [17]. Similarly, a study of children found that 41.2% of patients with persistent inflammation had arthritis, compared to 12.5% of those without subclinical inflammation [16]. In the present study, the prevalence of arthritis was found to be 14.6% in patients with subclinical inflammation, in comparison to 2.7% in the group without inflammation. Arthritis was also identified as an independent risk factor for subclinical inflammation in multivariate analysis.

A previous study reported that colchicine-resistant FMF patients, who reflect a high inflammatory burden, had earlier symptom onset and diagnosis, more frequent and prolonged attacks, and a higher prevalence of clinical features such as fever, erysipelas-like erythema, arthralgia/arthritis, myalgia, and abdominal and chest pain. These patients also had a higher prevalence of comorbidities, parental consanguinity, and exon 10 MEFV homozygous/compound heterozygous mutations. Multivariate analysis identified younger age at symptom onset, higher attack frequency, arthritis, chest pain, and the presence of two exon 10 MEFV mutations as the strongest predictors of colchicine resistance [23]. Similarly, our study found that prolonged disease duration, parental consanguinity, febrile attacks, and annual attack frequency were all significantly higher in the group with subclinical inflammation.

Biallelic exon 10 mutations, particularly M694V homozygosity, have been associated with a heightened inflammatory response and an increased risk of complications in FMF. Several studies have shown that these mutations may contribute to persistent inflammation even during attack-free periods, as evidenced by elevations in a wide range of inflammatory markers, including IL-18 and CRP [24]. Another study has highlighted the association of the M694V mutation with musculoskeletal involvement, including sacroiliitis, in FMF, as well as the clinical utility of inflammatory markers such as neutrophil-to-lymphocyte ratio (NLR), monocyte-to-lymphocyte ratio (MLR), and systemic immune-inflammatory index (SII) in reflecting subclinical inflammation and predicting disease-related complications [25].

In our cohort, the frequency of biallelic exon 10 mutations was significantly higher among patients with subclinical inflammation compared to those without, and this genetic pattern was identified as an independent predictor in a multivariate analysis. The ISSF score is an essential measure for evaluating the severity of FMF in patients [26]. However, there are limited studies in the literature examining the relationship between subclinical inflammation and the ISSF score. One study revealed a significant correlation between ISSF scores and subclinical inflammation: 64% of patients with subclinical inflammation had an intermediate ISSF score, compared to 41.5% of patients without subclinical inflammation. Furthermore, 29.6% of patients with subclinical inflammation had a severe ISSF score, compared to only 2.4% of those without (*p* < 0.001) [17]. Similarly, in the present study, 53.9% of patients with subclinical inflammation had an ISSF score of ≥ 3 (indicating moderate to severe disease severity), whereas this rate was 16.4% in the group without subclinical inflammation. In multivariate analysis, a high ISSF score was identified as an independent risk factor for subclinical inflammation. Other parameters such as age at disease onset, presence of fever, attack frequency, presence of arthritis, duration of disease, and carrying biallelic exon 10 mutations were found to be the most important predictors of subclinical inflammation.

A previous study showed that arterial stiffness increased in children with FMF and suggested that chronic subclinical inflammation could lead to early endothelial dysfunction and serve as an indicator of future cardiovascular risk [27]. Furthermore, it is well known that persistent subclinical inflammation is clinically relevant because it may contribute to cumulative inflammatory burden and long-term complications such as organ damage and amyloidosis. Therefore, the early detection and management of subclinical inflammation are crucial. In such cases, timely modification of treatment regimens, including augmentation of colchicine dosage, switching between colchicine formulations, or escalation to biologic therapy when indicated, may be imperative for effective disease management.

Our study demonstrates that nearly half of FMF patients with subclinical inflammation achieved remission following colchicine dose optimization or formulation change, whereas the remaining required biologic therapy, predominantly anti-IL-1 agents. As recent EULAR recommendations highlight, escalation to biologics should be considered early in colchicine-resistant patients to prevent irreversible organ damage and systemic complications [5]. However, before transitioning to IL-1 blockade therapy, colchicine adherence should be thoroughly assessed. Clinical practice indicates that in many cases, a delay in colchicine dose or missing even a single dose can trigger FMF attacks. Some patients may experience attacks or persist with inflammation, even at therapeutic doses of colchicine. In these cases, increasing the colchicine dose to the maximum tolerated dose may be effective. If disease control is not achieved despite this, biologic agents should be considered [28]. The high response rate to IL-1 blockade in our patients (*n* = 43, 87.7%) aligns with recent evidence supporting the central role of IL-1 in FMF pathophysiology and its efficacy in colchicine-resistant or subclinical inflammation cases [1]. In particular, non-response to classical therapies was observed in a small subgroup of patients with significant comorbidities such as CNO, IBD, and SAPHO, suggesting that overlapping autoinflammatory conditions may reduce therapeutic efficacy or increase the need for treatment. This reinforces the need for personalized treatment strategies and comorbidity screening in FMF patients with persistent inflammation despite standard therapy.

This study has several limitations. First, its retrospective single-center design may limit the generalizability of the findings. Therefore, larger prospective multicenter studies are needed to validate our results and to better define the long-term clinical implications of subclinical inflammation in pediatric FMF.

In conclusion, subclinical inflammation is not uncommon in pediatric FMF patients and should be considered an essential disease state in its entirety. Our results suggest that the early onset of disease, the presence of biallelic exon 10 mutations, and arthritis, as well as high ISSF scores, may be associated with persistent inflammation, and higher doses of colchicine were required to control inflammation in almost half of the patients with subclinical inflammation. For the remaining patients, this was not sufficient, and biological agent therapy was required and effective. The high response rate to IL-1 inhibitors supports the fundamental role of these agents in the management of subclinical disease. Although biological agents are effective, their use may be restricted in some countries due to limitations in access, high cost, and health policy regulations. Therefore, there remains a clear need for the development of cheaper, more accessible, and orally administered therapeutic alternatives. However, the presence of concomitant autoinflammatory diseases may adversely affect the response to treatment, necessitating a multidisciplinary and personalized treatment approach. Early recognition of subclinical inflammation, close monitoring, and implementation of individualized treatment strategies are crucial to prevent irreversible complications and improve long-term outcomes in this vulnerable patient group.

## Data Availability

Data are available to the public when a reasonable request is made.

## References

[1] Lancieri M, Bustaffa M, Palmeri S, Prigione I, Penco F, Papa R et al (2023) An update on familial Mediterranean fever. Int J Mol Sci 24(11):9584. 10.3390/ijms24119584 37298536 10.3390/ijms24119584 PMC10253709

[2] Aksentijevich I, Centola M, Deng Z, Sood R, Balow JE, Wood G et al (1997) Ancient missense mutations in a new member of the RoRet gene family are likely to cause familial Mediterranean fever. Cell 90(4):797–807. 10.1016/s0092-8674(00)80539-5 9288758 10.1016/s0092-8674(00)80539-5

[3] Bernot A, Clepet C, Dasilva C, Devaud C, Petit JL, Caloustian C et al (1997) A candidate gene for familial Mediterranean fever. Nat Genet 17(1):25–31. 10.1038/ng0997-25 9288094 10.1038/ng0997-25

[4] Chae JJ, Aksentijevich I, Kastner DL (2009) Advances in the understanding of familial Mediterranean fever and possibilities for targeted therapy. Br J Haematol 146(5):467–478. 10.1111/j.1365-2141.2009.07733.x 19466978 10.1111/j.1365-2141.2009.07733.x PMC2759843

[5] Ozen S, Sağ E, Oton T, Gül A, Sieiro Santos C, Bayraktar D et al (2025) EULAR/PReS endorsed recommendations for the management of familial Mediterranean fever (FMF): 2024 update. Ann Rheum Dis 84(6):899–909. 10.1016/j.ard.2025.01.028 40234174 10.1016/j.ard.2025.01.028

[6] Yalçinkaya F, Özen S, Özçakar ZB, Aktay N, Çakar N, Düzova A et al (2009) A new set of criteria for the diagnosis of familial Mediterranean fever in childhood. Rheumatology (Oxford) 48(4):395–398. 10.1093/rheumatology/ken509 19193696 10.1093/rheumatology/ken509

[7] Ben-Zvi I, Livneh A (2011) Chronic inflammation in FMF: markers, risk factors, outcomes and therapy. Nat Rev Rheumatol 7(2):105–112. 10.1038/nrrheum.2010.181 21060333 10.1038/nrrheum.2010.181

[8] Celkan T, Çelik M, Kasapçopur Ö, Özkan A, Apak H, Ocak S et al (2005) The anemia of familial Mediterranean fever disease. Pediatr Hematol Oncol 22(8):657–665. 10.1080/08880010500278681 16251171 10.1080/08880010500278681

[9] Zung A, Barash G, Zadik Z, Barash J (2006) Familial Mediterranean fever and growth: effect of disease severity and colchicine treatment. J Pediatr Endocrinol Metab 19(2):155–160. 10.1515/jpem.2006.19.2.155 16562589 10.1515/jpem.2006.19.2.155

[10] Düzova A, Özaltın F, Özon A, Beşbaş N, Topaloğlu R, Özen S et al (2004) Bone mineral density in children with familial Mediterranean fever. Clin Rheumatol 23(3):230–234. 10.1007/s10067-004-0874-y 15168151 10.1007/s10067-004-0874-y

[11] Lachmann HJ, Şengül B, Yavuzşen TU, Booth DR, Booth SE, Bybee A et al (2006) Clinical and subclinical inflammation in patients with familial Mediterranean fever and in heterozygous carriers of MEFV mutations. Rheumatology (Oxford) 45(6):746–750. 10.1093/rheumatology/kei279 16403826 10.1093/rheumatology/kei279

[12] Kısaoğlu H, Baba O, Kalyoncu M (2023) Genotype-phenotype associations of children with familial Mediterranean fever in a cohort consisting of M694V mutation and ımplications for colchicine-resistant disease. J Clin Rheumatol 29(4):207–213. 10.1097/RHU.0000000000001953 36870084 10.1097/RHU.0000000000001953

[13] Bayram MT, Çankaya T, Bora E, Kavukçu S, Ülgenalp A, Soylu A et al (2015) Risk factors for subclinical inflammation in children with familial Mediterranean fever. Rheumatol Int 35(8):1393–1398. 10.1007/s00296-015-3227-z 25669438 10.1007/s00296-015-3227-z

[14] Demirkaya E, Acikel C, Hashkes P, Gattorno M, Gül A, Özdoğan H et al (2016) Development and initial validation of İnternational severity scoring system for familial Mediterranean fever (ISSF). Ann Rheum Dis 75(6):1051–1056. 10.1136/annrheumdis-2015-208671 26823530 10.1136/annrheumdis-2015-208671

[15] World Health Organization (WHO) *Growth reference data for 5–19 years*. Available at: https://www.who.int/tools/growth-reference-data-for-5to19-years/indicators/bmi-for-age Accessed 10 September 2025

[16] Korkmaz C, Özdogan H, Kasapçopur O, Yazıcı H (2002) Acute phase response in familial Mediterranean fever. Ann Rheum Dis 61(1):79–81. 10.1136/ard.61.1.79 11779767 10.1136/ard.61.1.79 PMC1753891

[17] Ozen S, Demirkaya E, Erer B, Livneh A, Ben-Chetrit E, Giancane G et al (2016) EULAR recommendations for the management of familial Mediterranean fever. Ann Rheum Dis 75(4):644–651. 10.1136/annrheumdis-2015-208690 26802180 10.1136/annrheumdis-2015-208690

[18] Babaoglu H, Armagan B, Bodakci E, Satis H, Atas N, Sari A et al (2021) Predictors of persistent inflammation in familial Mediterranean fever and association with damage. Rheumatology (Oxford) 60(1):333–339. 10.1093/rheumatology/keaa378 32778893 10.1093/rheumatology/keaa378

[19] Gezgin Yıldırım D, Esmeray Şenol P, Söylemezoğlu O (2022) Predictors of persistent inflammation in children with familial Mediterranean fever. Mod Rheumatol 32(4):803–807. 10.1093/mr/roab054 34918114 10.1093/mr/roab054

[20] Yalçınkaya F, Özçakar ZB, Tanyıldız M, Elhan AH (2011) Familial Mediterranean fever in small children in Turkey. Clin Exp Rheumatol 29(4 Suppl 67):S87-90 21813071

[21] Demir F (2020) The musculoskeletal system manifestations in children with familial Mediterranean fever. North Clin Istanb 7(5):438–442. 10.14744/nci.2020.96636 33163878 10.14744/nci.2020.96636 PMC7603850

[22] Yenigün S, Ayla AY, Yüzbaşıoğlu MB, Başpınar SN, Ergün S, Karabicek A et al (2024) Characteristics of arthritis in patients with familial Mediterranean fever. Intern Med J 54(11):1802–1808. 10.1111/imj.16495 39166838 10.1111/imj.16495

[23] Batu ED, Sener S, Arslanoglu Aydin E, Aliyev E, Bagrul I, Turkmen S et al (2024) A score for predicting colchicine resistance at the time of diagnosis in familial Mediterranean fever: data from the TURPAID registry. Rheumatology 63(3):791–797. 10.1093/rheumatology/kead242 37228026 10.1093/rheumatology/kead242 PMC10907807

[24] Chaaban A, Yassine H, Hammoud R, Kanaan R, Karam L, Ibrahim JN (2024) A narrative review on the role of cytokines in the pathogenesis and treatment of familial Mediterranean fever: an emphasis on pediatric cases. Front Pediatr 12. 10.3389/fped.2024.1421353 39132307 10.3389/fped.2024.1421353 PMC11310175

[25] Atik I, Atik S (2024) Relationship between sacroiliitis and inflammatory markers in familial Mediterranean fever. Rev Assoc Med Bras (1992) 70(5). 10.1590/1806-9282.20240068 38775516 10.1590/1806-9282.20240068 PMC11111121

[26] Sağ E, Akal F, Atalay E, Akça UK, Demir S, Demirel D et al (2020) Anti-IL1 treatment in colchicine-resistant paediatric FMF patients: real life data from the HELIOS registry. Rheumatology (Oxford) 59(11):3324–3329. 10.1093/rheumatology/keaa121 32306038 10.1093/rheumatology/keaa121

[27] Sav NM, Altinsoy HB, Turen B, Gokce A (2025) Arterial stiffness and subclinical inflammation in children with familial Mediterranean fever: a comprehensive analysis. Children 12(2):232. 10.3390/children12020232 40003334 10.3390/children12020232 PMC11854315

[28] Levinsky Y, Azani L, Shkalim Zemer V, Chodick G, Tal R, Harel L et al (2023) Adherence to colchicine prophylaxis among patients with familial Mediterranean fever treated with interleukin-1 inhibitors. Semin Arthritis Rheum 61. 10.1016/j.semarthrit.2023.152211 37201215 10.1016/j.semarthrit.2023.152211

